# PERCEPT Indoor Navigation System for the Blind and Visually Impaired: Architecture and Experimentation

**DOI:** 10.1155/2012/894869

**Published:** 2012-12-17

**Authors:** Aura Ganz, James Schafer, Siddhesh Gandhi, Elaine Puleo, Carole Wilson, Meg Robertson

**Affiliations:** ^1^Electrical and Computer Engineering Department, University of Massachusetts, Amherst, MA 01003, USA; ^2^Department of Public Health, University of Massachusetts, Amherst, MA 01003, USA; ^3^Massachusetts Commission for the Blind, Executive Office of Health and Human Services, Boston, MA 02111, USA

## Abstract

We introduce PERCEPT system, an indoor navigation system for the blind and visually impaired. PERCEPT will improve the quality of life and health of the visually impaired community by enabling independent living. Using PERCEPT, blind users will have independent access to public health facilities such as clinics, hospitals, and wellness centers. Access to healthcare facilities is crucial for this population due to the multiple health conditions that they face such as diabetes and its complications. PERCEPT system trials with 24 blind and visually impaired users in a multistory building show PERCEPT system effectiveness in providing appropriate navigation instructions to these users. The uniqueness of our system is that it is affordable and that its design follows orientation and mobility principles. We hope that PERCEPT will become a standard deployed in all indoor public spaces, especially in healthcare and wellness facilities.

## 1. Introduction

The World Health Organization (2010) reported that globally the number of people of all ages visually impaired is estimated to be 285 million, of whom 39 million are blind [1]. Based on data from the 2004 National Health Interview Survey, 61 million Americans are considered to be at high risk of serious vision loss if they have diabetes, had a vision problem, or are over the age of 65 [2]. According to the American Diabetes Association diabetes is the leading cause of blindness in persons ages 20–74. An estimated 12,000 to 24,000 people lose their sight each year because of diabetes [3]. The Veteran Administration estimates that by 2020, there will be over 1 million Veterans with significant visual impairment and legal blindness. In addition, some 13 percent of the evacuated wounded service members in Iraq and Afghanistan have suffered a serious eye injury of one type or another [4].

The blind and visually impaired encounter serious problems in leading an independent life due to their reduced perception of the environment. New environments pose a huge challenge for them to perceive their surroundings without seeking help from others. Current training programs for blind and visually impaired people require them to memorize a large amount of information for numerous points of interest (i.e., university, shopping malls, bus terminals, etc.) leading to an increase in personal frustration. It is commonly accepted that the incapability of moving freely and independently can hinder the full integration of an individual into society [5]. Blindness, like other disabilities, affects one's mobility and quality of life [6], especially when the vision loss occurs at a later stage of adulthood after a lifetime with functional vision [7, 8]. 

The blind and visually impaired users encounter serious health problems, especially if their visual impairment is a result of diabetes [3]. In addition to blindness, diabetes has many health issues associated with it such as bone infections, nerve damage, and kidney failure. Such health-related problems require them to frequently use hospitals and public health services. Unfortunately, though, at present they are prevented from using them on the same terms as others. Very few health care settings seem to pay attention to the access needs of blind people when designing the physical environment. Modern hospitals are increasingly large and complex organizations, in which little attention appears to be paid to wayfinding for blind people in these complex environments, and most would be impossible to negotiate independently. 

There has been research to provide navigation information to the blind and visually impaired users both indoors and outdoors [9–20]. While most of these systems cover a wide range of functions, the end devices are complex and expensive, and none of these studies employed orientation and mobility (O&M) principles at the core of the system design. Moreover, the only study that tested an indoor navigation system with blind and visually impaired users (only three users) was reported in [19]. Recently, Google Maps 6.0 announced that they will add indoor navigation for retail and transit environments [21]. It has a visual interface that includes an illustration map found at malls and airports. It provides the user approximate location (using WiFi and cellular and GPS technologies) and has no navigation function. Since the system is designed for sighted users, the map and user location do not have to be accurate. In case Google will improve their localization as well as map representation and will provide access to this information we will be able to integrate it in our system. Moreover, we see these developments as very positive for PERCEPT system since the blind community will start to get used to Google indoor technologies and will be willing to adopt PERCEPT system which provides affordable, accurate, and O&M-based navigation instructions. Moreover, by using Google data we can integrate large spaces into our system such as airports and transit environments. Currently, developers can only overlay data onto Google maps but cannot get access to their databases, information required to develop an indoor navigation system for the blind and visually impaired.

In this paper we introduce PERCEPT system that provides enhanced perception of the indoor environment using passive radio frequency identification (RFID) tags deployed in the environment, a custom-designed handheld unit and Smartphone carried by the user, and PERCEPT server that generates and stores the building information and the RFID tags deployment. When a user, equipped with PERCEPT glove and a Smartphone, enters a multistory building equipped with PERCEPT system, he/she scans the destination at the kiosk located at the building entrance. The PERCEPT system directs the user to his/her chosen destination using landmarks (e.g., rooms, elevator, etc.). PERCEPT is different from other systems in the following aspects: (1) the user carries a custom made handheld unit with small form factor and an Android-based phone, (2) the system builds upon O&M principles, and (3) it is the first indoor navigation system tested with 24 blind and visually impaired subjects (other systems were either tested with up to three visually impaired users or were tested with blindfolded sighted users).

The paper is organized as follows. PERCEPT system architecture is introduced in the next section. A sample scenario is presented in Section 3, and Section 4 describes PERCEPT trials.  Section 5 concludes the paper. 

## 2. System Architecture

**Figure 1:**
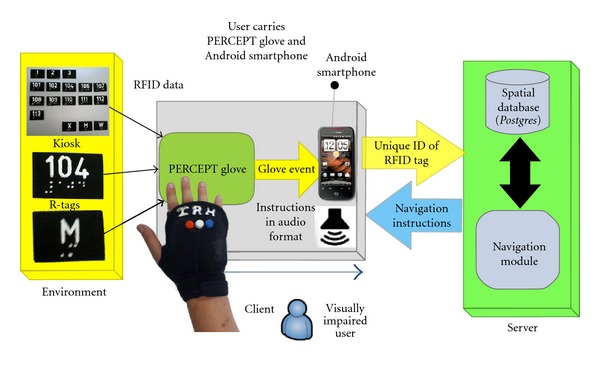
PERCEPT system overview.

PERCEPT system architecture which was briefly introduced in [22] consists of the following system components: the Environment, the PERCEPT glove and Android client, and the PERCEPT server (see Figure 1).

### 2.1. Environment

#### 2.1.1. R-Tags

 Passive RFID tags (R-tags) are deployed in the environment in strategic locations at 1.2 m height as dictated by the Americans with Disabilities Act (ADA) Guidelines. These guidelines also require the signage to include high contrast raised letters between 1.6 cm and 5 cm and embossed Braille [23]. R-tags are also embedded in kiosks located at specific points of interest such as the entrances/exits to a building, at the elevators, and at emergency exits. Granularity was the main reason behind selecting this technology. Proximity of 2-3 cm is required to transfer data from the R-tag into the reader. Other reasons for selecting these R-tags were their cost and the fact that they do not need any power source. On each R-tag we incorporate the room number in raised font and its Braille equivalent. 

#### 2.1.2. Kiosks

**Figure 2:**
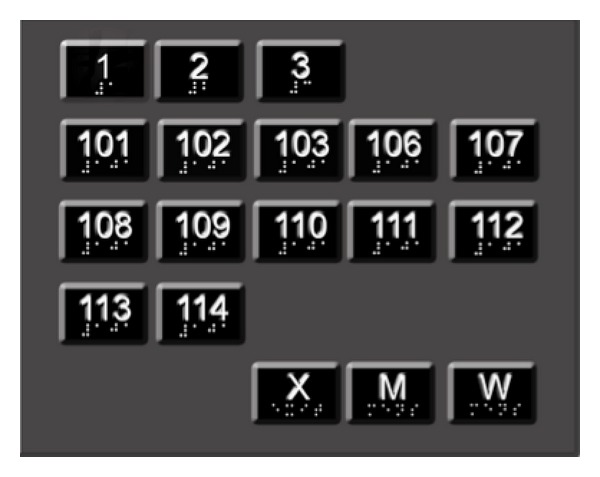
Kiosk design.

Kiosks are the junctions at which user's intention can be conveyed to the system. Kiosks are located at key points such as entrances/exits of the building, elevators, and emergency exits on each floor. As shown in Figure 2 kiosks contain R-tags that represent floor numbers and/or locations (rooms, restrooms denoted by M and W, and emergency exits denoted by X) in the building. By activating a specific R-tag, the user implicitly requests the navigation instructions to reach this destination (either a specific floor or a specific room number). 

### 2.2. PERCEPT Glove and Android Client

#### 2.2.1. PERCEPT Glove

**Figure 3:**
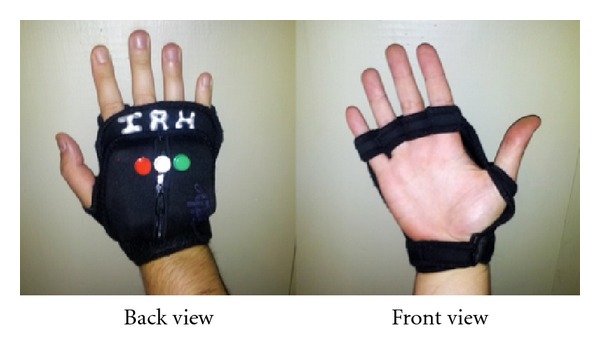
PERCEPT glove.

As shown in Figure 3 the glove allows the user free use of his hand as well as the ability to scan the R-tag. The user will first determine the requested R-tag (by using his fingers either through the embossed lettering on the R-tag or the Braille, or if the user has low functional vision, the user can identify visually through the high contrast large lettering) that represents the chosen destination. After the R-tag is determined, the user places his palm on top of the R-tag. The glove communicates the chosen destination represented in the R-tag using Bluetooth technology to the Android-based Smartphone. 

**Figure 4:**
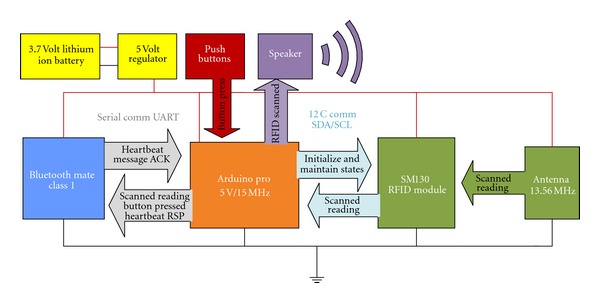
PERCEPT glove schematics.

Our PERCEPT glove system which is enclosed in a weight training glove includes (see Figure 4) Arduino microcontroller, RFID reader, antenna, Bluetooth chip, a set of buttons, speaker, rechargeable battery, and a power regulator. The Arduino microcontroller is used to keep track of all the events occurring during the interaction between the user wearing the PERCEPT glove and the environment. On scanning the R-tag, the RFID reader sends the R-tag data to the Arduino microcontroller. The Bluetooth chip is used to exchange data between the microcontroller and the Android Smartphone. The buttons on the PERCEPT glove provide users different options to interact with the PERCEPT system. Each of the buttons has a unique texture and can be identified through touch. The buttons represent different functionalities such as

1. *Help button (H)*: used for the return journey back to the kiosk.
2. *Replay/Rewind button (R)*: used to repeat previous instructions in case the user forgets them.
3. *Instructions button (I)*: instructions are broken in a set of easy-to-remember steps. After the user follows a set of instructions, he will press the Instruction button to get the next set of instructions.

#### 2.2.2. Android-Based Software

 Components of PERCEPT software implemented in the Android Smartphone are as follows.

1. *Bluetooth module*: this module is responsible for exchanging data (e.g., R-tag scan and Button press) between the Android Smartphone and the PERCEPT glove.
2. *PERCEPT application*: this application differentiates among various events, that is, *R-tag Scan Event, Help Button Press Event, Replay Button Press Event, and Instruction Button Press Event. *For R-tag Scan Event and Help Button Press Event, Unique Identifier of R-tag is sent to the PERCEPT Server over WiFi connection. Other events are processed locally, and the output is converted into an audio form using the Text to Speech Engine.
3. *Wi-Fi module*: Wi-Fi module is responsible for establishing Wi-Fi connection between the Android Smartphone and the PERCEPT server. The navigation instructions are received over the Wi-Fi connection from the PERCEPT server, which are then converted into an audio form using Text to Speech Engine.
4. *Text To Speech Engine*:as PERCEPT system is designed to assist the blind or visually impaired, the system interacts with the user in audio format. The Android Smartphone provides a built in Text To Speech Engine to convert the textual navigation information received from the server into audio format.

We investigated the power consumption of both the PERCEPT glove and the Android phone running PERCEPT application.

#### 2.2.3. PERCEPT Glove Power Consumption

The glove consumes on average 335 mA at 5 V. The current design of the glove is not optimized to save on power consumption. The Arduino, Bluetooth Module, and Passive RFID Module do not utilize their low power rest state and continually run in active mode. The glove uses a 1000 mAh lithium ion battery and will last on average 1 hour and 45 minutes of continuous usage.

#### 2.2.4. Android Phone Power Consumption

 Using the Android operating system battery utility on a Samsung Droid Charge phone, we collected statistics on the power usage of the screen, cellular communication, Bluetooth, WiFi, and other applications. 

On three separate days the Smartphone was as a personal phone (cellular phone call, browsing, email), and one hour during the day it was used with PERCEPT system. We started the experiments with a fully charged battery and took the statistics once the battery was almost fully discharged. On average over three days, we obtained the following statistics on the percentages of power consumption.

1. Activities during the day (94%): screen 50%, cellular communication 6%, Gmail 3%, Browser 3%, others applications and Operating System processes 32%.
2. PERCEPT activities (6%): application 2%, WiFi and Bluetooth which were only turned on for PERCEPT consumed 4%.

We conclude that the power consumption of PERCEPT application and associated communication activities (WiFi and Bluetooth) is minimal relative to other usages of the Smartphone.

### 2.3. PERCEPT Server

**Figure 5:**
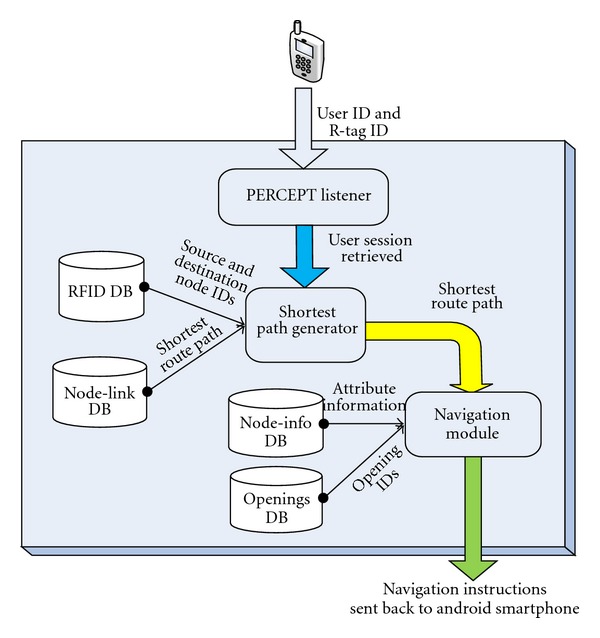
PERCEPT server architecture.

PERCEPT server architecture is depicted in Figure 5. Floor layouts of the entire building are constructed using *Quantum GIS* (geographic information system). Floor layout is a node-link structure (shapefile) with each node representing a room. Once the node-link structure (shapefile) is ready, it is mapped to the *Postgres *database. Every route in the node-link structure is associated with an attribute table which is a tabular depiction of the entire setup. This attribute table is used to generate the *Postgres* database table of a particular floor. The Navigation Module formulates navigation instructions after receiving the shortest path route from the *Postgres* database. It accesses *node_info* database to acquire information about each and every node in the shortest path route. The navigation instruction given to the user is of the following form: for example “*Please turn left and proceed along your right side wall for 4 doors and then you will reach your destination*.”

## 3. Sample Scenario

In this section we present a sample scenario to explain the flow of events.

**Figure 6:**
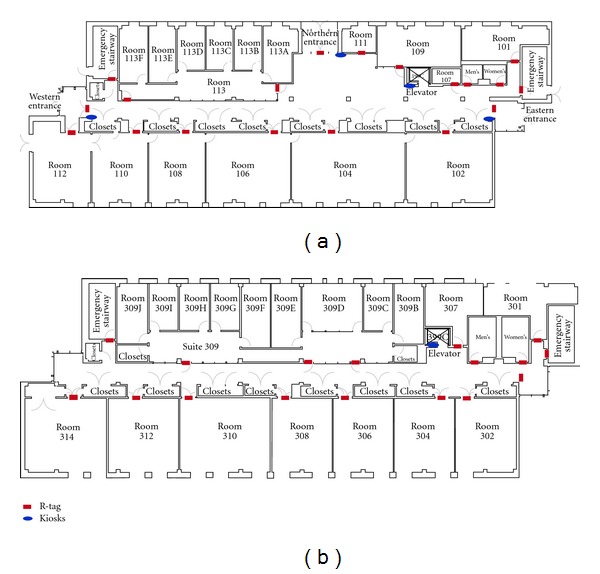
(a) 1st Floor structure of Knowles Engineering Building and (b) 3rd Floor structure of Knowles Engineering Building.

John is a freshman majoring in Computer Systems Engineering. He is trained by an O&M instructor on the use of the PERCEPT system. He stays in Knowlton Dormitory and wants to meet his advisor to discuss the courses he needs to take. His advisor's office is on 3rd floor (room number 312) in Knowles Engineering Building (see Figure 6). He calls campus Disability Services van for the ride to Knowles Engineering Building. John has a cane as well as a PERCEPT glove and an Android-based phone. It is assumed that John knows the destination room number and destination floor number. Here is John's journey.

1. *Designated drop-off point*: every building on campus has a designated drop-off point. Campus Disability Services van drops John at the designated entrance point (Eastern Exit in Figure 6(a)). Once he reaches Eastern Exit, he moves towards the kiosk located at Eastern Exit.
2. *Kiosk at the entrance*: the kiosk at Eastern Exit includes R-tags for every room on the 1st floor and one R-tag for other floors. John finds the R-tag that represents the 3rd floor using his finger and then uses his palm to scan the R-tag. He gets the following directions to reach the elevator on the 1st floor: “*Your Destination is on floor number 3, To reach your destination, Please turn right and proceed along your left side wall until you have reached one opening, Enter this opening. Once you reach there, please scan the card located on the Door*.” Route: (Eastern Exit > Room 102 > Room 107 > Elevator).
3. *Kiosk at the elevator on 1st floor*: once John reaches the elevator kiosk, he will scan the R-tag that belongs to the 3rd floor. Here he will get audio instructions informing him that he has reached the Elevator and he should proceed to 3rd floor (destination floor) using this elevator. John gets the following instructions: “*You have reached the Elevator of Floor number 1, Please use Elevator to proceed to Floor number 3. When you exit the elevator at Floor number 3, turn around and face elevator, the kiosk will be located to the left of the elevator door*.”
4. *Kiosk at the elevator on 3rd floor*: after John reaches the 3rd floor using the elevator (see Figure 6(b)), he will exit elevator, turn around to face the elevator door, and locate another kiosk to the left of the elevator door. Here, John will have to activate the R-tag that represents his destination room number that is, 312. Once he scans the desired destination R-tag with the PERCEPT glove, John gets the following directions to reach his destination room number (312): “*Your destination is Room 312, To reach your destination, Please turn Left and proceed along your Right side wall for 3 doors, Once you reach there, Scan the card located at the door*.” Route: Elevator > Room 309 C > Room 309 B > Room 309 A > Room 312.
5. *Any R-tag leads towards the destination*: In case John scans any R-tag on the floor he will be given directions to the destination he selected at the kiosk. For example, if John scans the R-tag at Room 306, he will get the following instructions: “*Please Turn right and proceed along your left side wall until you have reached the 3rd opening, Enter this opening and you will reach Room 312*.”
6. *Return journey*: after John finishes the meeting with his advisor, he wants to obtain the return instructions. He presses the HELP button on the PERCEPT glove. This will give him the following directions to reach the elevator on the 3rd floor: “*Your destination is Elevator. To reach your destination, Please put your back to the door and begin your return journey. Please proceed towards the opposite wall. After the user presses the instructions button the following instructions: Please turn Right and proceed along your left side wall until you reach the Elevator. Once you reach the elevator, Please scan the card belonging to your next destination*.”
7. *Kiosk at the elevator on 3rd floor*: once he reaches the kiosk, he will scan the R-tag that corresponds to 1st floor. John gets the following Instructions: “*You have reached the Elevator of floor number 3, Please use elevator to proceed to Floor number 1. When you exit the elevator at Floor number 1, turn around and face elevator, the kiosk will be located to the left of the elevator door*.”
8. *Kiosk at the elevator on 1st floor*: after reaching the elevator of floor number 1, John will scan the exit card on the kiosk. As he scans this card, he will get the following instructions: “*Your destination is EXIT door, To reach your destination, Please turn around and proceed towards the opposite wall. After the user presses the instructions button he gets the following instructions: Please turn Left and proceed along your right side wall until you reach 1st opening. Once you reach there, Please continue walking along the wall for 1 door and then you will reach the Exit door. Always scan the card located at the kiosk on the Right side of the Exit door before exiting the Floor number 1*.”
9. *Kiosk at eastern exit*: as John reaches the kiosk located at Eastern Exit, he scans the exit card, to convey to the system that he wants to leave the building. He gets the following instructions: “*You have reached the Exit door on floor number 1, Please open the Exit door and walk straight to go out of the building*.”

It is important to mention that in case the user gets lost he/she can scan any R-tag and obtain navigation instructions to the chosen destination.

## 4. PERCEPT Trials

PERCEPT results were briefly introduced in [24]. In this section we provide a detailed overview of our trials including the experimental design and quantitative and qualitative evaluations. 

We conducted two IRB approved phases of trials. In the first phase, 10 subjects provided feedback on ease of maneuvering around a building using PERCEPT system. Feedback from this first round of testing led to improvements in hardware (ruggedized it), changes in the way the trials were conducted (we adopted one-on-one trials as opposed to group trials), and changes in the delivery of the navigation instructions. A second phase of trials was conducted with 20 subjects. 

### 4.1. Phase I Trials

#### 4.1.1. Population

We had a diverse subject population as reported in Table 1. All 10 users received O&M training at some point during their onset of visual impairment or blindness. 

#### 4.1.2. Methods

**Figure 7:**
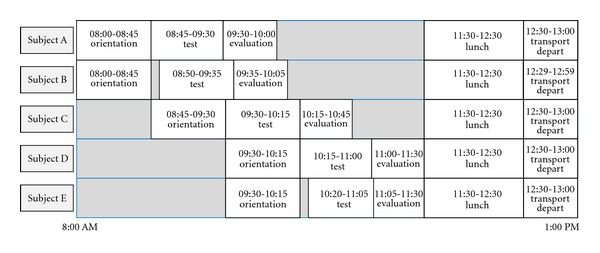
Phase I trials schedule.

 The 10 subjects were divided into two groups of five. These groups arrived on separate days to perform trials which followed three stages: orientation, test, and Evaluation (see Figure 7). Details of each stage in PERCEPT trials are provided in the following.

**Stage 1: Orientation**

The orientation session was given to two subjects. It took 45 minutes and was administered by an O&M instructor. In this stage the subjects are introduced to PERCEPT. First, we introduced PERCEPT hardware: PERCEPT glove, the kiosks, and the R-tags. PERCEPT system functionality was presented to the subjects by going through a system setup in a test area. A mock minitrial is done by asking the subject to navigate through a number of destinations in the test area using PERCEPT system. At any point the subject can stop and ask for help from the O&M instructor.

**Stage 2: Test**

**Figure 8:**
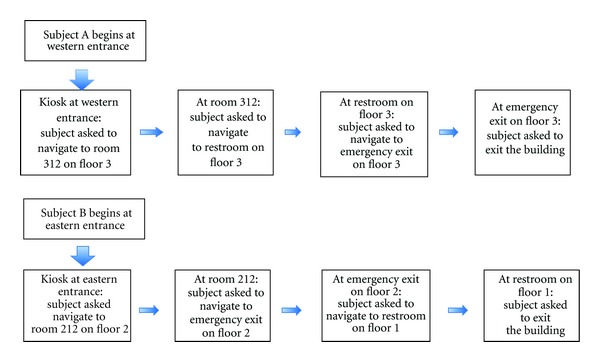
Test scenarios in Phase I trials.

In the test stage two subjects participated simultaneously. Each subject entered at a different entrance and was asked to navigate to eight destinations on different floors. In the destinations count we also include the return journey. The scenarios for each subject are depicted in Figure 8. There was one test proctor for each subject as well as an additional assistant that would videotape the trial (with subjects' consent). The test proctor would dictate the destinations that the subject needed to go to within the building. The proctor recorded the time it took for the subject to get to each destination. There were also “passerbyers” that would be walking randomly throughout the building. It is common practice for a visually impaired person to ask for assistance from those in the surrounding environment. So if the subject was unable to find the destination using PERCEPT, they could rely on a “passerbyer” to guide them or provide verbal directions to get to destination. The test proctor would record this occurrence on the testing form.

**Stage 3: Evaluation**

Each trial was videotaped (with the subject consent). The videotape is used for evaluating the system performance quantitative measures as described below.

*Stage 3.1: Quantitative Evaluation. *We used the following quantitative metrics.

NEI: navigation Efficiency Index is defined as the ratio between the length of *Actual Path Traveled and* the length of the *PERCEPT Path *(presumed to be optimal).

ACU: accuracy is defined as the ratio between the number of destinations reached by the subject and the total number of destinations determined by the trial. 

Average NEI for Phase I is 0.70.

Average ACU is 0.93. For 7 out of the 10 subjects, ACU = 1. Two subjects could not find one out of their 8 destinations due to PERCEPT system failure. One subject could not find three out of the eight destinations and asked for help.

*Stage 3.2: Qualitative Evaluation. *Each subject was asked the following qualitative questions regarding their experience with the PERCEPT system.

- Ease of trial: how easy was this trial for you to successfully complete?
- Level of confidence in self: how confident were you when the trial started, that you could accomplish the task successfully?
- Most difficult part of task: what was the most difficult part of the task?
- Easiest part of task: what was the easiest part of this task?
- Could this task be done in a crowd?
- How long did it take to learn how to use?
- How easy was PERCEPT to use?
- Likes/dislikes of PERCEPT.

The results were analyzed for trends to identify strong and weak points within the system as well as identify further suggested improvements. 70% of the subjects felt that PERCEPT trial was easy to complete, and 90% of the subjects felt confident after going into the trial after receiving orientation. 40% of the subjects expressed difficulty with a portion of the directions being too vague and trouble understanding the synthetic voice. 30% of the subjects felt that PERCEPT could be useful in the hall, but afterwards it was discovered that the subjects were not aware that an earpiece or headphones could be worn (during the trials PERCEPT was played out loud through the speaker of the device). 70% of the subjects said that it took less than 10 minutes to learn the device. 60% of the subjects thought PERCEPT was easy to use. The 40% that mentioned difficulties with the use of PERCEPT complained about the buttons on the glove.

It should be noted that in Phase I, there were 4 out of 10 subjects who reported either that they felt the device (i.e., PERCEPT glove) was uncomfortable and/or did not see the worth of the system to themselves personally. The 4 ranged in age (from 24 to 55) and education level; 3 of them were male, they were evenly divided between being partially sighted and completely blind, and all were only cane users. These subjects did not return for Phase II trials.

Changes made between Phase I and Phase II trials are as follows;

1. We ruggedized PERCEPT software and hardware (there were a number of technical failures in Phase I trials which we corrected).
2. Following the feedback from the subjects and staff participants, we decided to conduct one on one trials instead of group trials. We realized that the orientation should take more time and should be personalized to each subject. This is an expected conclusion since in practice, the O&M instruction is provided one-on-one due to the different learning abilities of each blind and visually impaired person.

### 4.2. Phase II Trials 

#### 4.2.1. Population

 We had a diverse subject population of 20 blind and visually impaired participants as reported in Table 2. Six subjects returned from Phase I. 

#### 4.2.2. Methods

 Each trial followed three stages: orientation, test, and evaluation. All stages were performed one on one with the subject and the test administrator.

**Stage 1: Orientation**

This stage is similar to the Orientation stage in Phase I trials with the important differences that it was conducted one-on-one and had no time limit. This stage took between 10 and 75 minutes.

**Stage 2: Test**

**Figure 9:**
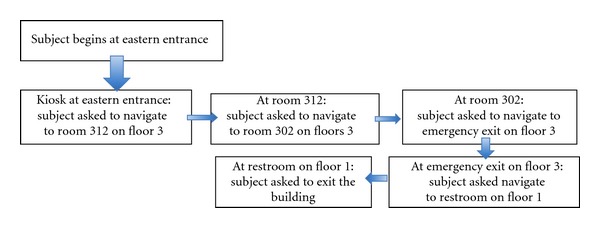
Test scenario in Phase II trials.

Each subject was asked to navigate to ten destinations within the building (same sequence of destinations is presented to each subject). The destinations (rooms, elevator, restroom, emergency exit, building exits) were located on the first and third floor of a typical classroom building (see Figure 9). The test administrator told the user the destinations, one at a time; that is, the next destination was given only after the current destination was successfully reached. During this stage the test administrator does not aid the subject with any navigational tasks. However, if the subject's safety was at all compromised, the trial administrator intervened on behalf of the well-being of the subject. If the subject was not able to find a destination, they could ask anyone in the environment for help; however this was recorded as a failure of the PERCEPT system.

**Stage 3: Evaluation**

Each trial was videotaped (with the subject's consent). The videotape is used for evaluating the system performance quantitative measures, NEI and ACU defined previously.

*Stage 3.1: Quantitative Evaluation.* The average NEI was 0.90 (in Phase I trials average NEI was 0.70). Investigators interpret this as an indication of the very high efficiency of the navigation instructions. 19 out of 20 users reached all the 10 destinations (ACU = 1). All of these users had previously received O&M instruction at some point since the onset of their visual impairment or blindness. The one user that did not receive O&M instruction was not able to use PERCEPT to the full extent.

**Figure 10:**
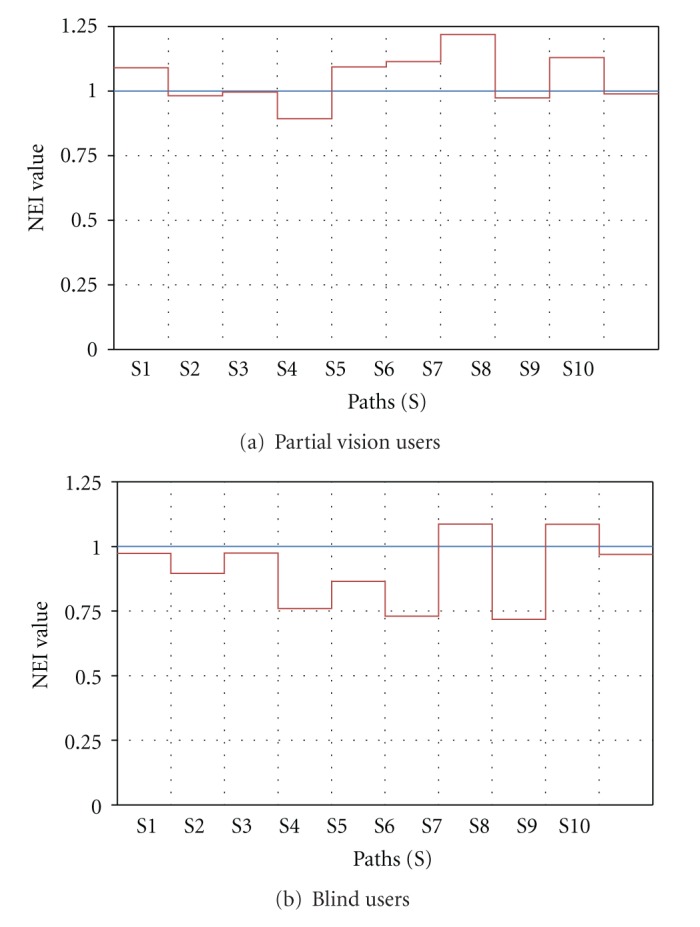
Navigation Efficiency Index, NEI, versus subpaths (S).

**Figure 11:**
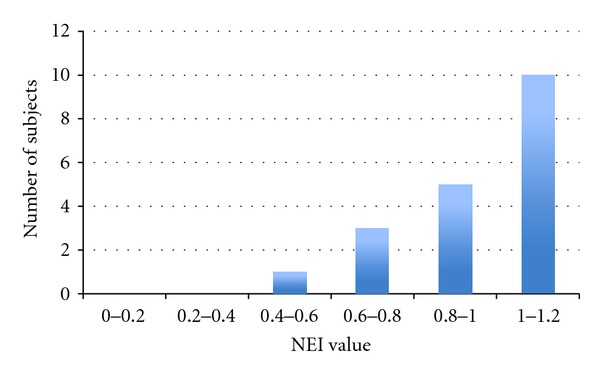
NEI distribution.

Figures 10(a) and 10(b) depict average NEI versus subpaths S (subpath is a portion of the path taken by the subject from source to destination) for partial vision and blind users, respectively. As expected, partial vision users performed better (i.e., have higher NEI) since they use visual cues. Notice that in Figure 10 for some subpaths NEI is higher than 1. This is due to the fact that PERCEPT navigation instructions follow the wall while users with partial vision can take shortcuts (do not always trail the wall). NEI distribution is depicted in Figure 11.

*Stage 3.2: Qualitative Evaluation.* Each subject was asked a series of qualitative questions regarding their experience with the PERCEPT system (see questions in Phase I description). The results were analyzed for trends to identify strong and weak points within the system, as well as identify further suggested improvements.

In Phase II, satisfaction was reported by all the subjects. 90% mentioned that PERCEPT system is intuitive, and 85% said that it provides independence and that they would use it. From Phase II, we found that females had slightly more self-reported difficulty with the use of the system. Subjects who had at least a college degree reported greater ease of use. 

The users suggested the following improvements. (1) Directions need to include proximity or given in feet/steps (5% of users). (2) Change instructions for those who have guide dogs (86% from dog users). (3) Provide options to adjust navigation instructions to user preferences—adjust voice pace—(60% of users). (4) Allow for abbreviated directions that should just mention left/right (15% of users). (5) Use of a Smartphone only (no additional hardware such as PERCEPT glove). 

## 5. Conclusions and Future Work

PERCEPT system that we designed, implemented, deployed, and successfully tested includes the following advantages: (1) the system design and the navigation instructions incorporate O&M principles and ADA guidelines, (2) the system is affordable to both the user and the building owners, and (3) the system is scalable to any size building.

When deployed in healthcare and wellness settings such as clinics, hospitals, and fitness centers, PERCEPT will enable independent access to these facilities. Therefore, PERCEPT will significantly improve the quality of life and health of the visually impaired community.

As a result of the trials presented previously we plan the following enhancements to PERCEPT.

1. We plan to replace the glove and the Smartphone by an NFC enabled Smartphone. Most of the current Android-based Smartphones include an NFC reader, and therefore PERCEPT glove is not required. We need to design Smartphone User Interface that follows Android accessibility features and allows for vision free operation.
2. Once Google will open the indoor APIs, we will integrate them in our system.
3. We plan to have one option of PERCEPT system that works on a Smartphone without the need for network connectivity to PERCEPT server. The user can download PERCEPT navigation directions before visiting a specific building. This option will enable us to use PERCEPT in areas that do not have network coverage. Moreover, this option will also reduce cellular data usage potentially reducing the cell phone bill.
4. Modify the navigation instructions as follows. (1) Directions will include proximity or will be given in feet/steps. (2) Include instructions for those who have guide dogs. (3) Provide options to adjust voice pace. (4) Allow for abbreviated directions that should just mention left/right.

## Figures and Tables

**Table 1:** Population for Phase I trials.

Field	Response	Number of subjects
Gender	Male	6
	Female	4

Race	Caucasian	8
	Hispanic	2

Age ( in years )	age < 20	0
	20 ≤ age < 30	2
	30 ≤ age < 40	2
	40 ≤ age < 50	1
	50 ≤ age < 60	3
	60 ≤ age < 70	1
	70 ≤ age < 80	0
	80 ≤ age < 90	1

Highest education level	GED/high school	2
	Some college	4
	College^+^	4

Members in household	1	4
	2	3
	3	1
	4	1
	5	0
	>5	1

Level of blindness	Blind	6
	Partial vision	4

Blind since birth	Yes	4
	No	6

Navigation aid	Cane	7
	Guide dog	3
	No mobility aid	0

Received O&M training	Yes	10
	No	0

**Table 2:** Population for Phase II trials.

Field	Response	Number of subjects
Gender	Male	8
	Female	12

Race	African American	3
	Caucasian	12
	Hispanic	5

Age (in years )	age < 20	2
	20 ≤ age < 30	2
	30 ≤ age < 40	2
	40 ≤ age < 50	2
	50 ≤ age < 60	9
	60 ≤ age < 70	2
	80 ≤ age < 90	1

Level of blindness	Blind	9
	Partial vision	11

Received O&M training	Yes	19
	No	1

Members in household	1	5
	2	9
	3	3
	4	1
	>5	2

Highest education level	GED	2
	High school	1
	Some college	6
	Undergraduate degree	9
	Masters degree	2

Blind since birth	Yes	5
	No	15

Navigation aid	Cane	10
	Guide dog	7
	No mobility aid	3
